# Indirect calorimetry identifies hypermetabolism associated with muscle wasting and increased risk of energy deficit in ICU patients

**DOI:** 10.1186/s13054-025-05695-y

**Published:** 2025-10-31

**Authors:** Janusz von Renesse, Moritz Karl Friedrich von Kessel, Florian Oehme, Johanna Kirchberg, Mikheil Kalandarishvili, Heiner Nebelung, Felix Merboth, Peter Mirtschink, Jürgen Weitz, Marius Distler, Hanns-Christoph Held, Jens-Peter Kühn, Ronny Meisterfeld

**Affiliations:** 1 Department of Visceral, Thoracic and Vascular Surgery, University Hospital and Faculty of Medicine Carl Gustav Carus, Technische Universität Dresden, 01307 Dresden, Germany; 2 National Center for Tumor Diseases (NCT), Partner Site Dresden, Heidelberg, Germany; 3 German Cancer Consortium (DKTK), Partner Site Dresden, German Cancer Research Center (DKFZ), Heidelberg, Germany; 4 Institute and Polyclinic for Diagnostic and Interventional Radiology, University Hospital Carl Gustav Carus, Technische Universität Dresden, Dresden, Germany; 5 Institute for Clinical Chemistry and Laboratory Medicine, University Hospital and Faculty of Medicine, Technische Universität Dresden, Dresden, Germany; 6 University Centre of Surgery Intensive Care Unit, University Hospital Carl Gustav Carus, Technische Universität Dresden, Dresden, Germany

**Keywords:** Muscle wasting, Muscle loss, Hypermetabolism, Hypermetabolizers, Hypermetabolic patients, Critical illness, Indirect calorimetry, Resting energy expenditure (REE), Sarcopenia, Cachexia, Intensive care unit (ICU)

## Abstract

**Background & aims:**

Muscle mass loss is a major contributor to morbidity and mortality in Intensive Care Unit (ICU) patients, but the role of metabolic state - particularly energy expenditure - in this process remains unclear. This study investigates the association between metabolic status and muscle mass loss in critically ill adults using indirect calorimetry and CT imaging assessed muscle quantification.

**Methods:**

In this observational study, adult ICU patients with at least two indirect calorimetry measurements and matched abdominal CT scans were included. Resting energy expenditure (REE) was measured by indirect calorimetry, and muscle mass was quantified as the cross-sectional area (CSA) of the posterior muscle group at the L3 vertebral level. Statistical analyses included regression modeling and group comparisons.

**Results:**

The observational study included 88 patients (*n* = 88), all of whom underwent at least two calorimetric measurements with corresponding CT scans, and 43 patients (*n* = 43) had at least three assessments. Persistently elevated normalized energy expenditure per kilogram of body weight (nREE) was independently associated with greater muscle loss. Patients classified as hypermetabolic by nREE experienced significantly more muscle wasting than those with lower metabolic activity. Hypermetabolism was associated with increased inflammatory markers, while sedation or agitation (RAAS) and higher level of consciousness (GCS) were not related to metabolic state.

**Conclusions:**

Persistent hypermetabolism in ICU patients is independently associated with accelerated muscle mass loss. Early identification of hypermetabolic patients using indirect calorimetry may enable targeted nutritional interventions to reduce muscle mass wasting and improve clinical outcomes.

## Background

Muscle mass loss presents a significant challenge in healthcare, particularly for critically ill patients in the ICU [1, 2]. In this setting, rapid muscle wasting contributes substantially to increased morbidity and mortality, severely complicating patient recovery and convalescence. ICU patients with trauma, surgery, or severe infections often develop stress-induced hypercatabolism, leading to rapid muscle breakdown [3]. Alarmingly, substantial muscle mass can be lost within a single week of ICU admission [4], underscoring the urgency of understanding and mitigating this process.

Although CT and MRI are gold standards for assessing muscle mass [5], determining metabolic status remains challenging. Critical illness states, such as those induced by infection, burns, malignancy, or major surgery, cause profound metabolic shifts – altering both energy substrate utilization (e.g. lipids, ketone bodies, or carbohydrates) and overall energy demands [6]. Traditional methods for estimating caloric needs, primarily based on predictive equations, often prove inaccurate compared to directly measured values in this population [7, 8].

The precise mechanisms driving muscle mass loss in critical illness remain incompletely elucidated. A prevailing hypothesis is that hypermetabolism accelerates muscle loss, particularly when muscle is catabolized over fat during stress [9, 10]. However, this critical hypothesis requires further empirical substantiation with robust clinical data.

A deeper understanding of the interplay between metabolic status and muscle catabolism could pave the way for individualized nutritional and therapeutic strategies, aiming to curtail muscle mass depletion and ultimately improve patient outcomes. Our current observational study aims to illuminate this topic by examining the association between metabolic status, precisely determined by indirect calorimetry, and objectively measured muscle mass loss in ICU patients.

## Materials & methods

In this study, we adhered to the ethical guidelines set forth in the Declaration of Helsinki. All measurements took place at the university center of surgery intensive care unit of the University Hospital Carl Gustav Carus at the University of Technology Dresden.

For our study, we sourced data from the database of our observational study, selecting patients who were admitted between July 30, 2019, and March 6, 2021. Participants were required to be above 18 years of age. Their intensive care unit stay, and hence survival, had to span a minimum of one week. Additionally, their fractional inspired oxygen (FIO2) had to be below a maximum of 70%. Furthermore, each included patient had to be subjected to at least two calorimetry measurements (REE-measurements) with an interval of no less than one week between them during his or her ICU stay. Coinciding with each calorimetry test, a CT scan of the abdomen had to be conducted for each REE measurement within the same week.

Calorimetry was performed by trained operators as part of standard clinical routine, diligently following established recommendations previously documented [11]. The Q-NRG + calorimeter by COSMED (Rome, Italy) was employed for this procedure. After a designated 20-minute resting period, indirect calorimetry (IC) was initiated. The initial 5 min of the measurements were utilized for stabilization and, as such, were excluded from the final analysis. Using the measured oxygen consumption (VO2) and carbon dioxide production (VCO2), the calorimeter computed the total energy expenditure (in kcal/day), as described elsewhere [12]. The precision and reliability of these measurements were ensured through regular maintenance and functional tests of the device, in line with manufacturer recommendations.

Predicted REE (pREE) was calculated using the Harris–Benedict equations as revised by Roza and Shizgal (1984) [13]:

$$\begin{aligned} \text{Male}:{\text{ }}\text{BMR}{\text{ }} &= {\text{ }}\left( {13.397{\text{ }} \times {\text{ }}\text{weight}{\text{ }}\left[ \text{kg} \right]} \right){\text{ }} + {\text{ }}\left( {4.799{\text{ }} \times {\text{ }}\text{height}{\text{ }}\left[ \text{cm} \right]} \right){\text{ }}\\&\quad-{\text{ }}\left( {5.677{\text{ }} \times {\text{ }}\text{age}{\text{ }}\left[ \text{y} \right]} \right){\text{ }} + {\text{ }}88.362\end{aligned} $$

$$\begin{aligned}\text{Female}:{\text{ }}\text{BMR}{\text{ }} &= {\text{ }}\left( {9.247{\text{ }} \times {\text{ }}\text{weight}{\text{ }}\left[ \text{kg} \right]} \right){\text{ }} + {\text{ }}\left( {3.098{\text{ }} \times {\text{ }}\text{height}{\text{ }}\left[ \text{cm} \right]} \right){\text{ }}\\&\quad-{\text{ }}\left( {4.330{\text{ }} \times {\text{ }}\text{age}{\text{ }}\left[ \text{y} \right]} \right){\text{ }} + {\text{ }}447.593\end{aligned} $$

We analyzed CT scans obtained during routine care on three multidetector scanners (SOMATOM Definition AS+, Edge, Force; Siemens Healthineers, Forchheim, Germany). Protocols were applied according to clinical indications (native vs. contrast-enhanced; slice thickness adapted as required). We extracted cross-sectional Digital Imaging and Communications in Medicine (DICOM) images at the level of the third lumbar vertebra (L3). This anatomical landmark was chosen because regional analysis of fat and fat-free tissue at the L3 level using CT has been shown to strongly predict whole-body fat and fat-free mass (*r* = 0.86–0.94; *p* < 0.001), making it a reliable indicator of whole-body muscle mass [5, 14].

We used semiautomatic segmentation with MedSeg (Oslo, Norway; accessed Dec 16, 2022) as described previously [15]. All segmentations were performed by a trained expert blinded to patient background and calorimetry results, and reviewed by a second observer to ensure consistency. Given that the Hounsfield unit (HU) ranges of muscle tissue overlap with those of adjacent tissues and edema, a fully automated segmentation process was deemed unsuitable. This concern was particularly pertinent due to the prevalence of widespread severe edema in our study population - a condition frequently observed in critically ill ICU patients [16].

The following HU thresholds were used to differentiate tissue types:

Muscle tissue: −29 to + 150 HU.

Intramuscular adipose tissue: −190 to −30 HU [5].

**Fig. 1:**
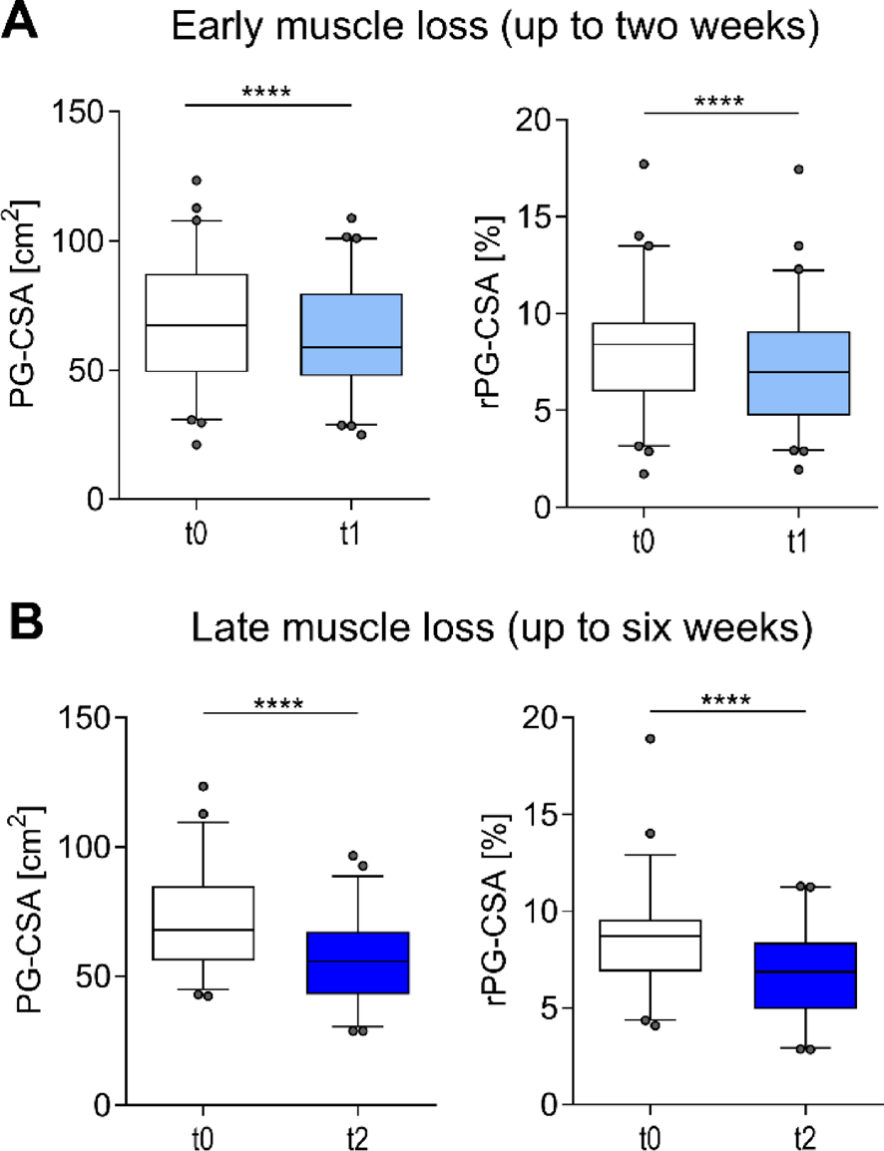
Muscle loss observed during ICU short- and long-term admission. (**A**) Early muscle loss: Comparison of PG-CSA (cm²), and rPG-CSA (%) between the first baseline (t0) and second measurement (t1) within two weeks of ICU stay (*n* = 60). (**B**) Long-term muscle loss: Comparison of PG-CSA (cm²) and rPG-CSA (%) between baseline (t0) and later follow-up (t2) beyond two weeks but within six weeks (*n* = 52). **** *p* < 0.0001

It is well established that the cross-sectional area CSA of the psoas and paraspinal muscles serves as a reliable surrogate for total muscle mass [17]. This correlation is sufficiently strong that clinical indices, such as the psoas muscle mass index (PMI), exclusively utilize the paraspinal muscle group [18, 19]. Because fluid overload during ICU care may disproportionately enlarge ventral compartments, we additionally calculated the relative PG-CSA (rPG-CSA) (Fig. 1A, B).

For each image, we calculated the CSA of the paravertebral and paraspinal muscle group (hereafter referred to as the posterior group [PG]). The PG-CSA comprised the psoas major, erector spinae, and quadratus lumborum muscles. Additionally, a comprehensive segmentation of the entire tissue region was performed to account for all relevant anatomical structures. Extraneous materials such as catheters, pads, tables, and linens were either manually excluded or naturally fell outside the specified HU range for the total CSA, defined as − 300 to + 2000 HU.

The segmented masks were used to compute the total-CSA, and the PG-CSA, expressed in square centimeters (cm²). We calculated the rPG-CSA (%) as a proportion of the total-CSA to provide a relative measure of muscle mass.

Statistical evaluations were conducted utilizing the R statistical platform (version 4.3.1, R Foundation for Statistical Computing, www.r-project.org). For graphical representations, we employed GraphPad Prism v8 (GraphPad Software, Inc, La Jolla, CA, USA) and, where relevant, the rempsyc package (version 0.1.6) within the R environment. To assess demographic and clinical data, we applied the Shapiro-Wilk-test for normal distribution and subsequently used either the Student’s t-test or the Mann-Whitney-U-test for group comparisons, based on data distribution. Categorical data were presented in terms of absolute numbers and proportions (percentages), and the χ2 test or exact Fischer test was utilized for comparison, contingent on the sample size. For linear regression analyses, we leveraged the capabilities of the rempsyc package (version 0.1.6) in R. In the multivariable analysis, we applied linear regression models with muscle loss (ΔrPG-CSA) as the dependent variable and energy expenditure (kcal/kg/day) as the main predictor, adjusted for CRP, leukocyte count, SOFA score, RASS, and GCS.

### Declaration of generative AI and AI-assisted technologies in the writing process

AI tools (ChatGPT-4) were only used to improve readability; all content was reviewed and verified by the authors.

## Results

### Baseline characteristics of included individuals

The study included 88 patients, all of whom underwent at least two calorimetric measurements with corresponding CT scans, and 43 had at least three assessments. The cohort’s mean age was 67.2 (± 12.0) years, with 33 females and 55 males (37.5% and 62.5% respectively). Patients had an average ICU stay of 38.5 (± 24.2) days and an overall hospital stay of 44.4 (± 29.1) days. The mean Body Mass Index (BMI) was 27.6 (± 5.76) kg/m², with 29.5% (*n* = 26) classified as obese (BMI ≥ 30 kg/m²) (Table 1).

**Table 1:** Baseline patient characteristics

Parameter	*n* = 88 (SD, or %)
Sex	
Female	33 (37.5%)
Male	55 (62.5%)
Age	67.2 (12.0)
BMI	27.6 (5.76)
Obese (BMI > 30):	
No	62 (70.5%)
Yes	26 (29.5%)
REE [kcal/24 h]	1687 (370)
Normalized REE [kcal/24 h/kg]	21.3 (4.18)
SAPS	44.3 (12.3)
ICU stay duration [days]	38.5 (24.2)
Total hospital stay [days]	44.4 (29.1)
Major ICU diagnosis	
Neurosurgical	15 (17.0%)
Traumatologic/Orthopedic	12 (13.6%)
Vascular	9 (10.2%)
Visceral Surgery	52 (59.1%)

Values are n (%) or mean (SD) respectively

Regarding primary department of treatment, 52 patients (59.1%) were from the Department of Visceral Surgery, 9 (10.2%) from Vascular Surgery, 12 (13.6%) from Traumatology and Orthopedics and 15 (17.0%) from Neurosurgery (Table 1). The average REE was 1687 kcal/24 h (± 370), corresponding to 21.3 kcal/kg/day (± 4.18) when normalized to total body weight (Table 1). At admission, the mean Simplified Acute Physiology Score (SAPS) was 44.3 (± 12.3) (Table 2).

**Table 2:** Clinical characteristics and of hypermetabolic (high) and non-hypermetabolic (low) patients

Parameter	high (*n* = 22)	95% CI	low (*n* = 21)	95% CI	*P*
Overall					
Sex					1.000
Female	8 (36.36%)	17.2–59.3	8 (38.10%)	18.1–61.6	
Male	14 (63.64%)	40.7–82.8	13 (61.90%)	38.4–81.9	
Age	66.18 (10.77)	61.4–71.0	64.86 (12.88)	59.0–70.7.0.7	0.717
BMI	25.93 (4.22)	23.7–27.7	29.91 (4.85)	28.3–32.6	0.007
Obese (BMI > 30):					0.090
No	17 (77.27%)	54.6–92.2	10 (47.62%)	25.7–70.2	
Yes	5 (22.73%)	7.8–45.4	11 (52.38%)	29.8–74.3	
SAPS Score (adm.)	44.18 (12.92)	38.4–49.4	42.95 (10.39)	37.4–47.4	0.732
ICU stay duration [days]	45.07 (23.60)	38.0–52.2.0.2	48.01 (34.81)	37.3–58.7	0.749
Total hospital stay [days]	49.27 (27.02)	41.2–57.4	56.38 (42.79)	43.2–69.6	0.521
ICU Discharge Modality:					0.126
Deceased	6 (27.27%)	10.7–50.2	9 (42.86%)	21.8–66.0	
Rehabilitation Facility or Hospital	16 (72.73%)	49.8–89.3	10 (47.62%)	25.7–70.2	
Standard Discharge or Home Care	0 (0.00%)	0.0–15.4.0.4	2 (9.52%)	1.2–30.4	
At 2nd and 3rd calorimetry measurement	high (*n* = 44)	95% CI	low (*n* = 42)	95% CI	
mREE/pREE [%]	136.52% (22.03)	129.8–143.2.8.2	107.13% (18.44)	101.4–112.9.4.9	< 0.001
SOFA Score	13.04 (4.40)	11.2–14.9	11.65 (3.79)	10.3–13.0	0.222
GCS	7.18 (3.29)	5.9–8.5	8.97 (3.45)	7.7–10.2	0.043
RASS	−2.50 (1.49)	−3.0 - −2.0	−2.19 (1.76)	−2.7 - −1.6	0.381
Lactate	1.58 (1.22)	1.2–1.9	1.42 (0.89)	1.1–1.7	0.486
CRP [mg/L]	157.10 (88.87)	130.1–184.1.1.1	128.67 (67.31)	107.7–149.6.7.6	0.097
Leucocyte count [GPt/L]	17.40 (7.94)	15.0–19.8.0.8	14.17 (8.29)	11.6–16.8	0.071
Nutrition [kcal/24 h]	1346.20 (564.60)	1214–1541	1367.45 (434.46)	1232–1502	0.845
Percent kcal received of REE	72.01 (27.72%)	63.6–80.4	87.87 (36.72%)	76.4–99.3	0.027
Protein [kcal/24 h]	271.38 (149.51)	225.4–317.4.4.4	313.77 (139.21)	270.4–357.1.4.1	0.180
Protein % kcal received of REE	14.84 (8.17)	12.4–17.3	20.02 (9.42)	17.1–23.0	0.008
Indications for CT					0.403
Hemorrhage/Vascular	8 (18.18%)	8.2–32.7	6 (14.29%)	5.4–28.5	
Infectious/Septic Work-up	18 (40.91%)	26.3–56.8	12 (28.57%)	15.7–44.6	
Ischemia/Thrombosis/Embolism	4 (9.09%)	2.5–21.7	3 (7.14%)	1.5–19.5	
Obstruction/Passage disorders	3 (6.82%)	1.4–18.7	1 (2.38%)	0.1–12.6	
Post-intervention follow-up	1 (2.27%)	0.1–12.0	3 (7.14%)	1.5–19.5	
Postoperative complications	10 (22.73%)	11.5–37.8	7 (40.48%)	25.6–56.7	

Values are n (%) or mean (SD) respectively

### Rapid and sustained muscle mass loss in ICU patients

To assess early muscle loss within two weeks in the ICU, we compared the first and second PG-CSA from patients (*n* = 60) whose second measurement occurred within two weeks of the first (Fig. 1A and B). To account for fluid overload, relative muscle area (rPG-CSA) was also computed (Fig. 1A, B right). A significant decline in muscle mass was observed within two weeks for both PG-CSA (69.03 cm² ± 23.70 cm² vs. 62.85 cm² ± 20.92 cm², *p* < 0.001), and rPG-CSA (8.13% ± 3.04% vs. 7.13% ± 2.91%, *p* < 0.001) (Fig. 1A).

To investigate the extended trajectory of muscle mass loss, we analyzed patients with at least two measurements separated by more than two but less than six weeks (*n* = 52). As anticipated, a stronger decline in muscle mass was observed in both PG-CSA (70.96 cm² ± 20.13 cm² vs. 55.87 cm² ± 15.87 cm², *p* < 0.001) and in rPG-CSA (8.63% ± 2.71% vs. 6.84% ± 2.35%, *p* < 0.001) (Fig. 1B), These findings confirm substantial muscle loss within two weeks of ICU stay, with continued progression thereafter.

### Association between normalized energy expenditure and muscle mass loss

To investigate the relationship between metabolic status and muscle mass loss, we assessed patients with at least three paired calorimetry and CT examinations (*n* = 43). Muscle mass loss was quantified as the difference in rPG-CSA (ΔrPG-CSA) between the first and last measurements.

**Fig. 2:**
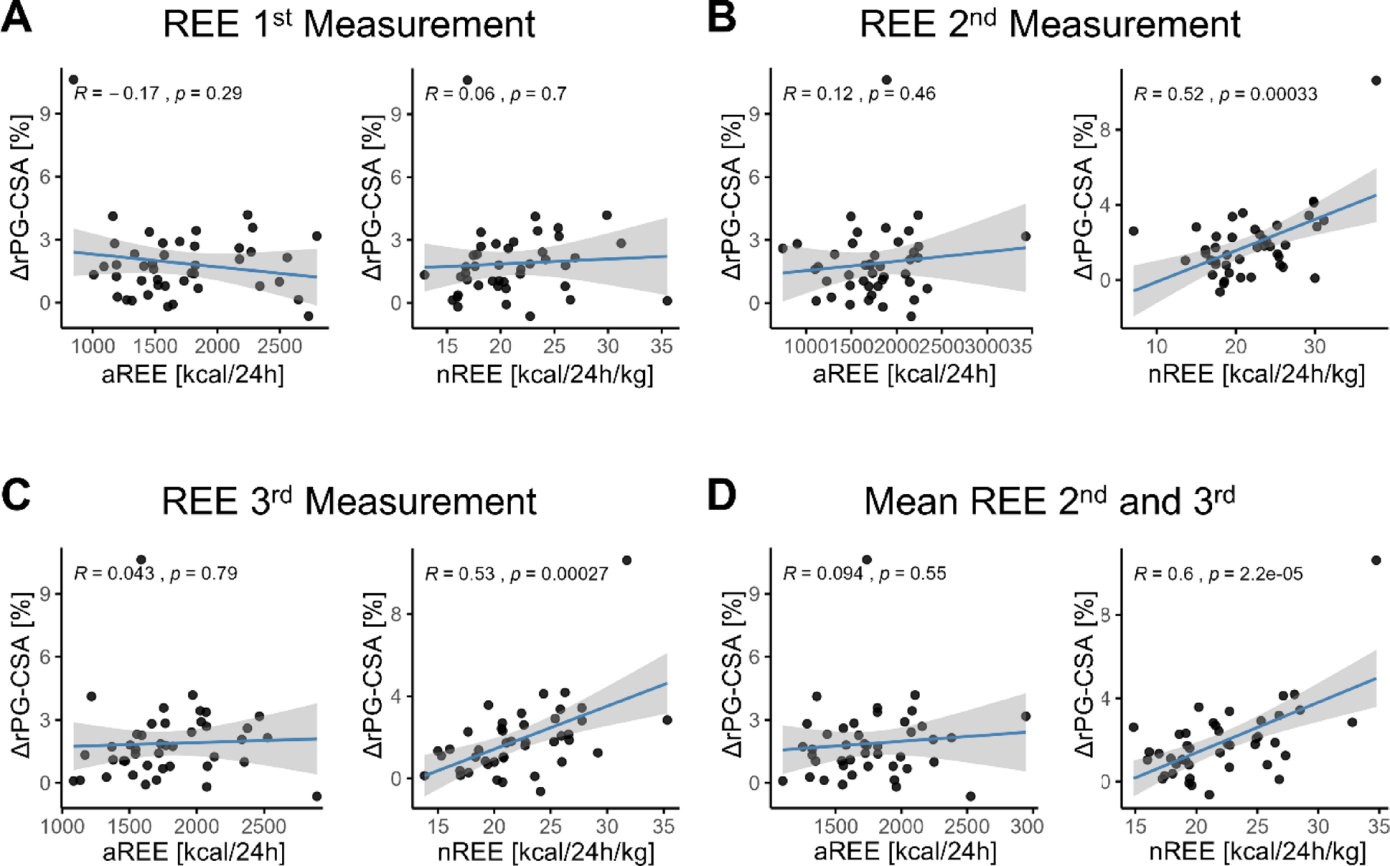
Sustained hypermetabolism as a driver of muscle reduction. Linear regression analysis between nREE (kcal/kg/day) and muscle mass loss (ΔrPG-CSA, %) across three calorimetry timepoints. (**A**) First measurement (t0): No significant correlation between nREE and ΔrPG-CSA. (**B**, right) Second measurement nREE (t1), (**C**, right) third measurement nREE (t2), (**D**, right) and the average nREE (mean of second and third measurements) show a strong correlation with muscle mass loss. (**A**-**D** left) No correlation between aREE and muscle mass loss at any timepoint

We performed linear regression analysis between ΔrPG-CSA and 24-hour REE, both in absolute terms (aREE) and normalized to body weight (nREE). At the first, second and third measurement timepoints (Fig. 2A and C).

During the early phase of the ICU stay (first measurement), no significant correlation was found between aREE or nREE and muscle mass loss (aREE: *r* = −0.17, *p* = 0.285; nREE: *r* = 0.06, *p* = 0.701) (Fig. 2A). However, at the second and third measurements, a significant positive correlation was found between the nREE and muscle mass loss (2nd : *r* = 0.52, *p* < 0.001; 3rd : *r* = 0.53, *p* < 0.001) (Fig. 2B and C, right) a correlation not observed for absolute REE (2nd : *r* = 0.12, *p* = 0.461, 3rd : *r* = 0.04, *p* = 0.786) (Fig. 2B and C, left).

To assess the impact of prolonged energy turnover, we calculated the average nREE and aREE from the second and third measurements. While average aREE showed no significant association with muscle mass loss (*r* = 0.09, *p* = 0.548), average nREE demonstrated an even stronger correlation (*r* = 0.60, *p* < 0.001) (Fig. 2D). To further account for potential confounding, we performed multivariable linear regression adjusting for systemic inflammation (CRP and leukocyte count), organ dysfunction (SOFA), sedation depth (RASS), and level of consciousness (GCS). Increased nREE remained strongly associated with increased muscle loss (β = 0.247, 95% CI 0.139–0.355, *p* < 0.001), whereas CRP, leukocyte count, SOFA score, RASS, and GCS were not significant independent predictors (Supplementary Table 1). These findings support an independent association between hypermetabolism and muscle catabolism beyond systemic inflammation, illness severity, and sedation depth. Together, these findings indicate that persistently elevated energy expenditure per kg body weight in ICU patients is associated with increased muscle mass loss.

### Hypermetabolism is associated with increased muscle loss and inflammation

**Fig. 3:**
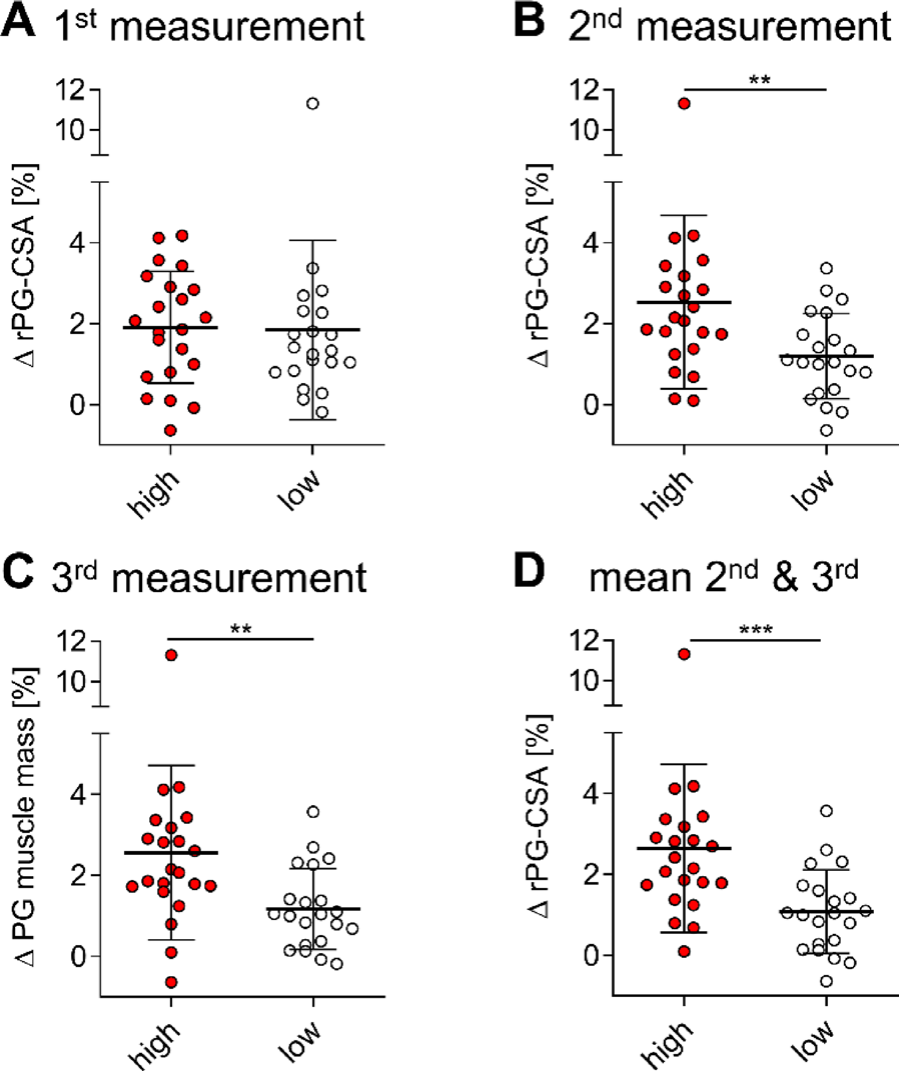
Hypermetabolic patients experience significantly greater muscle mass reduction. Patients classified as high or low metabolizers based on median nREE. Comparison of muscle mass loss (ΔrPG-CSA, %) between groups across timepoints: (**A**) First measurement: No significant difference. (**B**) second measurement, (**C**) third measurement, (**D**) and mean of second and third measurements: Hypermetabolic patients show significantly greater loss. *p < 0.05, **p < 0.01, ***p < 0.001

To investigate differences in muscle mass loss between patients with high and low energy turnover (hereafter referred to as “hypermetabolic”), we divided patients with three or more calorimetric measurements into two groups based on the median nREE (Fig. 3A and D). This classification was applied to the first (Fig. 3A), second (Fig. 3B), third (Fig. 3C) measurements, and the mean of the second and third measurements (Fig. 3D).

At the first REE measurement, no significant difference in muscle mass loss was observed between high and low metabolizers (ΔrPG-CSA: 1.91% vs. 1.85% ± 2.21%, *p* = 0.341) (Fig. 3A). However, in later measurements (second and third), muscle mass loss was significantly greater in the hypermetabolic group compared to low-metabolizers (ΔrPG-CSA : 2nd : 2.53% ± 2.15% vs. 1.20% ± 1.05%, *p* = 0.006; 3rd : 2.56% ± 2.16% vs. 1.17% ± 0.10%, *p* = 0.003) (Fig. 3B and C). Comparing groups based on the median of the mean energy expenditure from the 2nd and 3rd measurements, hypermetabolic patients showed a notably larger decline in muscle mass (ΔrPG-CSA : 2.65% ± 2.08% vs. 1.08% ± 1.03%, *p* = 0.0006) (Fig. 3D).

We then explored associations between hypermetabolism and baseline patient characteristics, clinical scores, or laboratory results corresponding to the respective timepoints. To examine this, we compared the long-term high and low metabolizer groups, as defined by the median of the mean energy expenditure (grouped as in Fig. 3D) (Table 2). First, we assessed whether our group distribution aligned with definitions based on increased measured REE (mREE) relative to predicted REE (pREE). Hypermetabolic patients exhibited a markedly higher mREE/pREE [%] ratio compared to non-hypermetabolic patients (136.52% ± 22.03% vs. 107.13% ± 18.44%, *p* < 0.001), supporting the validity of our group classification (Table 2). No significant differences were found in sex distribution (female/male; high: 8/14 (36.36%, and 63.64%); low: 8/13 (38.10%, and 61.90%) (*p* = 1.000) or age (high: 66.18 years ± 10.77; low: 64.86 years ± 12.88, *p* = 0.717) (Table 2).

Interestingly, a higher proportion of overweight individuals was observed in the low metabolizer group (high: *n* = 5 (22.7%); low: *n* = 10 (11 (52.4%)), albeit this difference did not reach statistical significance (*p* = 0.09). Yet, the BMI of non-hypermetabolic patients was discernibly lower when set against hypermetabolic patients (high: 25.93 kg/m² ± 4.22 kg/m², vs. low: 29.91 kg/m² ± 4.85 kg/m², *p* = 0.007).

To assess whether the observed effect was merely a result of differing weight distributions, we categorized patients into Obese and Non-Obese groups and repeated the analysis within each group. Notably, within these subgroups, high metabolizers consistently exhibited greater muscle mass decline compared to their low metabolizer counterparts (ΔrPG-CSA: non-Obese: high: 2.93% ± 2.42%, vs. low: 1.37% ± 1.12%, *p* = 0.021; Obese: high: 1.76% ± 1.22%, vs. low: 0.90% ± 0.78%, *p* = 0.106), although significance was not reached in the Obese subgroup, what may be attributed to the smaller subgroup sample size (Supl. Figure 1).

No significant differences in disease severity at admission, as indicated by SAPS scores, or in clinical status at the second and third timepoints, as reflected by SOFA scores, were observed between the groups (SAPS: 44.18 ± 12.92 vs. 42.95 ± 10.93, *p* = 0.732; SOFA: 13.04 ± 4.40 vs. 11.65 ± 3.79, *p* = 0.222) (Table 2).

Differences in energy expenditure could potentially be influenced by variations in patient activity or level of consciousness [20]. To address this, we compared the Glasgow Coma Scale (GCS) and the Richmond Agitation-Sedation Scale (RASS) between groups. While RASS (RASS: − 2.50 ± 1.49 vs. − 2.19 ± 1.76, *p* = 0.381) scores did not differ, hypermetabolic patients exhibited significantly lower GCS scores (GCS: 7.18 ± 3.29 vs. 8.97 ± 3.45, *p* = 0.043), suggesting reduced consciousness despite increased energy expenditure.

We next assessed whether laboratory markers of inflammation and lactate levels were associated with hypermetabolism. Although lactate levels did not differ between groups (1.58 mmol/L ± 1.22 mmol/L vs. 1.42 mmol/L ± 0.89 mmol/L, *p* = 0.486), both CRP (157.10 mg/L ± 88.87 mg/L vs. 128.67 mg/L ± 67.31 mg/L, *p* = 0.097) and leukocyte counts (17.40 G/L ± 7.94 G/L vs. 14.17 G/L ± 8.29 G/L, *p* = 0.071) were elevated in hypermetabolic patients, with differences approaching statistical significance. To better understand the potential drivers of the elevated inflammatory markers, we compared CT indications between groups. The overall distribution of indications did not differ significantly (*p* = 0.403); however, infectious/septic work-up was the most frequent reason for CT imaging and was numerically more common among hypermetabolic patients (40.91% [*n* = 18] vs. 28.57% [*n* = 12]), although this difference was not significant. Postoperative complications represented the second most frequent indication, with a higher proportion observed in the low-metabolizer group (22.73% [*n* = 10] vs. 40.48% [*n* = 17]) (Table 2).

Notably, the proportion of calories received relative to REE was significantly lower in hypermetabolic patients (72.01% ± 27.72% vs. 87.87% ± 36.72%, *p* = 0.027), despite similar caloric supply over 24 h (1346.20 kcal/24 h ± 564.60 kcal/24 h vs. 1367.45 kcal/24 h ± 434.46 kcal/24 h, *p* = 0.845) (Table 2). The absolute protein intake did not differ between groups (271.38 ± 149.51 kcal/24 h vs. 313.77 ± 139.21 kcal/24 h, *p* = 0.180). However, the proportion of protein contributing to resting energy expenditure was significantly lower in hypermetabolic patients (14.84 ± 8.17% vs. 20.02 ± 9.42%, *p* = 0.008) (Table 2).

In summary, our findings suggest that hypermetabolism in ICU patients appears linked to ongoing inflammatory processes and this elevated metabolic demand, if unmet, contributes to caloric deficits and muscle wasting.

## Discussion

Our study demonstrates that persistent hypermetabolism, assessed by serial indirect calorimetry, is independently associated with accelerated muscle mass loss in ICU patients. This finding provides direct evidence linking increased energy expenditure to measurable muscle depletion, supporting a long-standing hypothesis in critical care.

Predictive equations are often inaccurate in the ICU [25]. Our findings confirm that persistently elevated energy demands are best measured by indirect calorimetry. While hypermetabolic states leading to muscle breakdown have been described in cancer cachexia and severe burns [21], our work extends this paradigm to the ICU setting. To our knowledge, this is among the first studies to demonstrate that ongoing hypermetabolism, rather than a single early elevated measurement, correlates with increased muscle wasting throughout a patient’s ICU course. Of note, hypermetabolic patients in our cohort had an average mREE/pREE ratio of 136.5%, compared to 107.1% in the low group. This highlights the pronounced hypermetabolic state in this patient population undergoing repeated CT imaging, a phenomenon similarly reported in burn and pancreatitis patients [22–24]. Although a universally accepted definition of hypermetabolism is lacking, our findings are consistent with previously proposed cut-offs ranging from 110% to 130% of predicted REE [24–26].

Importantly, our results reveal an “at-risk” patient subgroup within the ICU – patients exhibiting sustained hypermetabolism – who experienced more rapid muscle decline. These findings challenge the classical “ebb–flow–recovery” model of critical illness metabolism [27, 28], suggesting that hypermetabolism may persist outside these phases, particularly in the presence of systemic inflammation. These findings align with emerging data that link failure to transition between metabolic phases or ongoing catabolism with poor ICU outcomes [29].

Inflammation may causally contribute to both hypermetabolism and catabolism, and in our cohort hypermetabolic patients showed higher CRP and leukocyte counts. Inflammation as surrogate or hypermetabolism itself may represent an independent risk factor for muscle loss. Future studies should examine whether inflammatory biomarkers can help identify patients most likely to benefit from repeated calorimetric monitoring.

Hypermetabolic patients received fewer calories relative to measured REE, with a lower protein contribution, likely worsening catabolism. Caloric deficits often persist beyond ICU discharge, underscoring the prolonged vulnerability to malnutrition [30]. Whether increasing caloric intake can meaningfully prevent muscle loss in this subgroup remains to be tested and should be a focus of future interventional trials [31, 32]. Importantly, a recent systematic review found insufficient high-quality evidence to demonstrate that enteral overfeeding causes adverse clinical outcomes [33]. Only minor effects were observed, including increased insulin needs and gastrointestinal intolerance [33]. This suggests that increasing caloric intake may represent a safe strategy.

ESPEN and ESICM guidelines recommend the use of indirect calorimetry where available [34], but implementation is often constrained by resources. In practice, our findings support a targeted approach in case of resource restrictions: baseline IC followed by trigger-based re-measurement (infection/deterioration, major therapeutic/physiologic shifts, or sustained under/overfeeding), rather than universal routine IC for all ICU patients.

There are several limitations to this study. The requirement for paired CT scans led to an overrepresentation of surgical and oncology patients, as these groups are more likely to undergo serial imaging. This opens the question of whether the association between infection, hypermetabolism, and muscle loss is also present in ICU patients less prone to inflammation. Nevertheless, infectious complications are a common driver of prolonged ICU stay across patient populations, suggesting that our findings may be relevant beyond the surgical/oncology-focused cohort, even if they do not fully capture the entire ICU spectrum. Additionally, the monocentric design might limit generalizability; multicenter studies are needed to validate these results. Although our analysis focuses on muscle loss as an intermediate endpoint, prior ICU studies have linked greater early muscle loss to higher mortality, longer ventilation and ICU stay, and poorer functional recovery; our finding that hypermetabolism associates with accelerated muscle loss provides a biologically plausible pathway that warrants prospective evaluation with prespecified hard outcomes.

In conclusion, our study establishes persistent hypermetabolism as a clinically relevant risk factor for muscle mass loss among ICU patients. These findings underscore the urgent need for targeted metabolic monitoring and the development of precision nutritional therapies in critical care.

## Data Availability

The datasets used and analyzed during the current study are available from the corresponding author on reasonable request.

## Abbreviations

BMI: Body Mass Index
CRP: C-Reactive Protein
CSA: Cross-Sectional Area
CT: Computed Tomography
DICOM: Digital Imaging and Communications in Medicine
FiO_2_: Fractional Inspired Oxygen
GCS: Glasgow Coma Scale
HU: Hounsfield Unit
ICU: Intensive Care Unit
IC: Indirect Calorimetry
MRI: Magnetic Resonance Imaging
mREE: Measured Resting Energy Expenditure
nREE: Normalized Resting Energy Expenditure
pREE: Predicted Resting Energy Expenditure
PG: Posterior Group
PG-CSA: Posterior Group Cross-Sectional Area
PMI: Psoas Muscle Index
RAAS: Richmond Agitation Sedation Scale
REE: Resting Energy Expenditure
SAPS: Simplified Acute Physiology Score
SOFA: Sequential Organ Failure Assessment
